# Synthesis, Crystal Study, and Anti-Proliferative Activity of Some 2-Benzimidazolylthioacetophenones towards Triple-Negative Breast Cancer MDA-MB-468 Cells as Apoptosis-Inducing Agents

**DOI:** 10.3390/ijms17081221

**Published:** 2016-07-29

**Authors:** Hatem A. Abdel-Aziz, Wagdy M. Eldehna, Hazem Ghabbour, Ghada H. Al-Ansary, Areej M. Assaf, Abdullah Al-Dhfyan

**Affiliations:** 1 Department of Applied Organic Chemistry, National Research Center, Dokki, Giza 12622, Egypt; 2 Department of Pharmaceutical Chemistry, Faculty of Pharmacy, Egyptian Russian University, Badr City, Cairo 11829, Egypt; wagdy2000@gmail.com; 3 Department of Pharmaceutical Chemistry, College of Pharmacy, King Saud University, P.O. Box 2457, Riyadh 11451, Saudi Arabia; ghabbourh@yahoo.com; 4 Department of Medicinal Chemistry, Faculty of Pharmacy, Mansoura University, Mansoura 35516, Egypt; 5 Department of Pharmaceutical Chemistry, Faculty of Pharmacy, Ain Shams University, Abbassia, Cairo 11566, Egypt; ghada.mohamed@pharma.asu.edu.eg; 6 Department of Biopharmaceutics and Clinical Pharmacy, Faculty of Pharmacy, The University of Jordan, Amman 11942, Jordan; areej_assaf@ju.edu.jo; 7 Stem Cell & Tissue Re-Engineering Program, Research Center, King Faisal Specialized Hospital & Research Center, MBC-03, P.O. Box 3354, Riyadh 11211, Saudi Arabia; pharm101696@hotmail.com

**Keywords:** synthesis, X-ray, anti-proliferative, breast cancer MDA-MB-468 cells, apoptosis

## Abstract

On account of its poor prognosis and deficiency of therapeutic stratifications, triple negative breast cancer continues to form the causative platform of an incommensurate number of breast cancer deaths. Aiming at the development of potent anticancer agents as a continuum of our previous efforts, a novel series of 2-((benzimidazol-2-yl)thio)-1-arylethan-1-ones **5a**–**w** was synthesized and evaluated for its anti-proliferative activity towards triple negative breast cancer (TNBC) MDA-MB-468 cells. Compound **5k** was the most active analog against MDA-MB-468 (IC_50_ = 19.90 ± 1.37 µM), with 2.1-fold increased activity compared to 5-fluorouracil (IC_50_ = 41.26 ± 3.77 µM). Compound **5k** was able to induce apoptosis in MDA-MB-468, as evidenced by the marked boosting in the percentage of florecsein isothiocyanate annexin V (Annexin V–FITC)-positive apoptotic cells (upper right (UR) + lower right (LR)) by 2.8-fold in comparison to control accompanied by significant increase in the proportion of cells at pre-G1 (the first gap phase) by 8.13-fold in the cell-cycle analysis. Moreover, a quantitative structure activity relationship (QSAR) model was established to investigate the structural requirements orchestrating the anti-proliferative activity. Finally, we established a theoretical kinetic study.

## 1. Introduction

Pertaining to the frequency of diagnosis worldwide, breast cancer is regarded as the second most frequently diagnosed cancer and the most frequently diagnosed tumor among women. Also, it is regarded as the fifth leading cause of cancer mortality [1]. In 2012, an estimated 1.67 million newly diagnosed breast cancer cases and 522,000 breast cancer deaths occurred worldwide [1]. The etiology of breast cancer is still unknown, although different risk factors have been established—to name just a few, first-degree relative’s breast cancer family history, mammographic density, benign breast disease, younger age at menarche, low parity, older age at first birth, older age at menopause, high postmenopausal body mass index, low premenopausal body mass index, and endogenous hormone levels have been established as risk factors for breast cancer [2,3].

Breast cancer is regarded as a diverse group of diseases with multiple intrinsic tumor subtypes that have various treatment modalities and long-term survival probabilities. The immunohistochemical expression of the estrogen receptor (ER), progesterone receptor (PR), and human epidermal growth factor receptor-2 (HER-2) forms the platform of characterization of clinically defined breast cancer subtypes [4]. In approximately 15%–20% of globally diagnosed breast cancers, the tumors do not express ER, PR, or HER-2. Such malignancies are designated as triple-negative breast cancer (TNBC) [5].

In the current medical era, TNBC, among all the breast cancer subgroups, has stood out as the greatest clinical challenge as these tumors have no clinically validated molecularly targeted therapy, are prevalent in younger women, associated with the worst prognosis, and often relapse rapidly. Also, TNBC are highly proliferative, poorly differentiated, often grade III carcinomas, genetically unstable, and preferentially metastasize to the brain and lungs [6,7,8,9,10]. Therefore, there is a critical need to develop potent and effective novel therapies to improve the outcomes of TNBC treatment.

**Figure 1:**
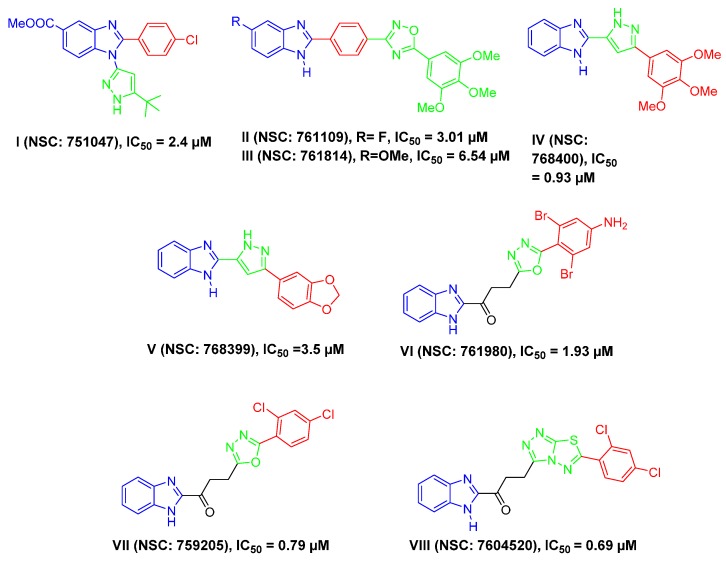
Structures of some reported benzimidazoles **I**–**VIII**, by other research groups, with anti-proliferative activity against triple-negative breast cancer MDA-MB-468 cells [22,23,24,25,26,27].

Because of their similarity to some naturally occurring nucleotides and their existence in several naturally occurring compounds, benzimidazole derivatives possess a wide range of biological activities and therapeutic effects [11,12,13]. In the field of medicinal chemistry, benzimidazole represents a highly privileged scaffold and has been copiously explored as an anti-proliferative agent targeting different breast cancer cells [14,15,16,17,18,19,20,21]. Surveying the literature revealed that different benzimidazole-based scaffolds were developed with significant activity toward the TNBC MDA-MB-468 cells in the anticancer drug screening program of the U.S. National Cancer Institute (NCI), according to their applied protocol against full NCI 60 human cell lines panel (Figure 1).

Many researchers have reported the utility of 2-aryl benzimidazole derivatives as anti-proliferative agents against TNBC. Attaching a heterocyclic moiety, 5-*tert*-butyl-1*H*-pyrazol-3-yl, in position 1 and an aryl moiety, 4-chlorophenyl, in position 2 of benzimidazole core resulted in compound **I** (NSC: 751047) with good anti-proliferative activity against MDA-MB-468 (IC_50_ = 2.4 µM) [22] (Figure 1), while substitution of position 2 of 5-flouro and 5-methoxybenzimidazole with 1,2,4-oxadiazole moiety through a phenyl ring afforded compounds **II** and **III**, respectively, (NSC: 761109 and 761814) with IC_50_ values of 3.01 and 6.54 µM, respectively, against MDA-MB-468 [23] (Figure 1). Moreover, introduction of different aryl groups through a pyrazole linker at position 2 of the benzimidazole core, as in compounds **IV** and **V** (NSC: 768400 and 768399), achieved significant efficacy against MDA-MB-468 (IC_50_ values of 0.93 and 3.5 µM, respectively) [24] (Figure 1). Interestingly, linking different aryl moeities, through variable heterocyclic groups, via a three-atom linker, namely a propan-1-one group to position 2 of the benzimidazole core led to compounds **VI**–**VIII** (NSC: 761980, NSC: 759205 and NSC: 7604520) with low or sub-micromolar anti-proliferative activity against MDA-MB-468 (IC_50_ = 1.93, 0.79 and 0.69 µM, respectively) [25,26,27] (Figure 1).

In addition, we recently introduced an efficacious benzimidazole-based scaffold as for development of potent antitumor agents that prove to have anti-proliferative activity not only toward the cancer stem cells but also toward the bulk of tumor cells of the colon HT-29 cell line [28]. The design of such a scaffold relies on linking different aryl or heteroaryl groups to position 2 of the benzimidazole core through a thio ethan-2-one linker. From the findings reported above, we came to the conclusion that linking the 2-position of benzimidazole scaffold to a terminal aryl or heteroaryl group directly or via variable spacers—an aryl, a heteroaryl, or a propan-1-one group—affords promising molecules that have significant anti-proliferative activity against TNBC.

**Figure 2:**
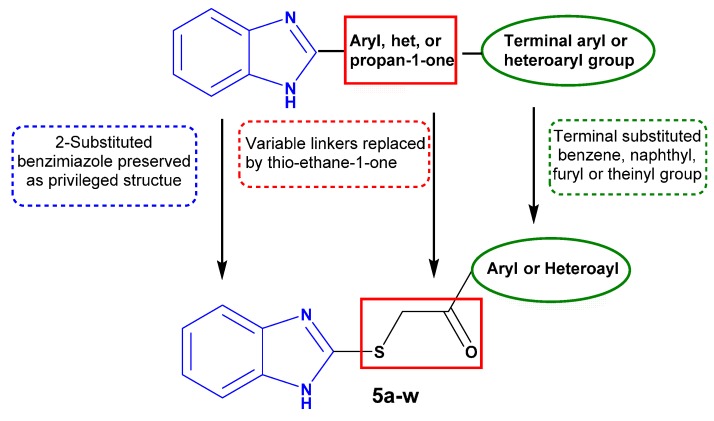
Structure-based design of target benzimidazoles **5a**–**w** as anti-triple-negative breast cancer (TNBC) agents.

Regarding these points and as a continuation of our research program on the design and synthesis of effective antitumor candidates [29,30,31,32,33,34,35], it was thought worthwhile to extend our investigations around our study [28] to probe for benzimidazole derivatives having anti-proliferative activity towards TNBC. Our structure-based design was three-fold: (i) preserving benzimidazole structure with subsitution at 2-position; (ii) maintaining a terminal lipophilic group; and (iii) establishing a three-atom thio ethane-1-one linker to afford more flexibility for the designed molecules (Figure 2). Thus, the present work reports the synthesis of benzimidazoles **5a**–**w** and their in vitro anti-proliferative activity against the TNBC MDA-MB-468 cell line. Moreover, the most active member in this study, **5k**, was selected to be further investigated regarding its effects on cell cycle progression and potential apoptotic effect in the MDA-MB-468 cells, to acquire perception of the mechanism of the anti-proliferative activity of the prepared compounds. Eventually, a theoretical kinetic study was constituted.

## 2. Results

### 2.1. Synthetic Approach to Prepare the Target Derivatives

**Scheme 1:**
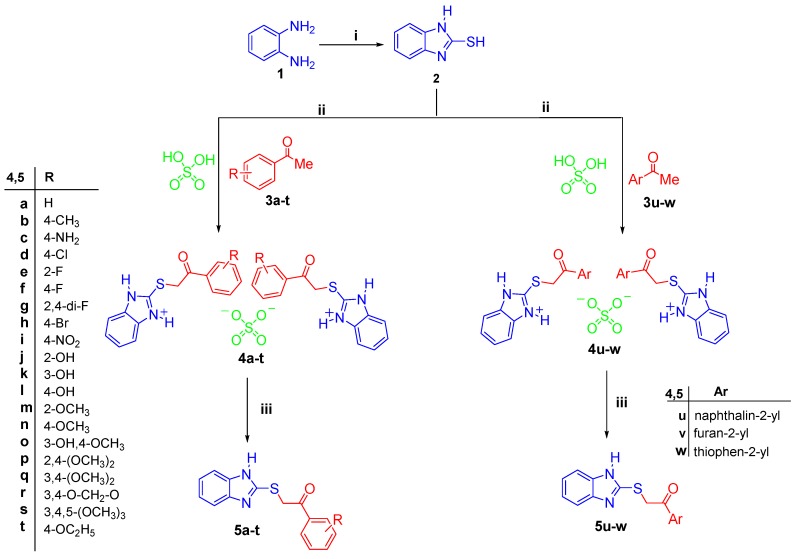
Synthesis of the target 2-((benzimidazol-2-yl)thio)-1-arylethan-1-ones **5a**–**w** and their corresponding sulfate salts **4a**–**w**. Reagents and conditions: (**i**) CS_2_/KOH/ethanol/reflux 2 h; (**ii**) Glacial acetic acid/reflux 0.5 h; (**iii**) Aqueous Na_2_CO_3_/r.t. (room temperature) 2 h.

The target compounds were prepared following our recently published procedure [28] via the reaction of compound **2** with different aromatic ketones **3a**–**w** in glacial acetic acid in the presence of two equivalents conc. H_2_SO_4_ to afford a quantitative yield from the sulfate salts **4a**–**w**. The prepared sulfate salts **4a**–**w** were subsequently neutralized to afford the target 2-((benzimidazol-2-yl)thio)-1-arylethan-1-ones **5a**–**w** in an excellent yield of 82%–96% (Scheme 1).

Infrared (IR) spectra for compounds **5a**–**w** displayed absorption bands attributable for the NH group in the range 3324–3460 cm^−1^, also a (C=O) band in the range of 1654–1690 cm^−1^. Also, their ^1^H-nuclear magnetic resonance (NMR) spectra displayed one singlet D_2_O-exchangeable signal due to the NH proton in the range of δ 12.52–12.80 ppm, whereas (–CH_2_–) protons appeared as singlet signals within δ 5.00 ppm. Furthermore, the ^13^C-NMR spectra of compounds **5a**–**w** showed signals resonating in the region δ 182.16–193.90 ppm due to the carbon of carbonyl group, whereas the carbons of the (–CH_2_–) group appeared in the region of δ 38.46–43.97 ppm.

### 2.2. Single Crystal Analysis of Compounds **4u** and **5v**

Crystals of compounds **4u** and **5v** were selected to analyze their single-crystal X-ray crystallographic after slow evaporation from solutions of ethanol. The instrument used is Bruker SMART APEX II D8 Venture diffractometer (Bruker, Karlsruhe, Germany) with graphite-monochromated Mo *K*α radiation (λ = 0.71073 Å) at 100 and 150 K, respectively. A direct method was applied to solve the structures that were subsequently refined with SHELXTL [36]. The positions of all the non-H-atoms were provided by E-maps. Using anisotropic temperature factors, the full-matrix least-squares refinement was carried out on *F*^2^’s for all non-H-atoms. Crystallographic data was deposited in the Cambridge Crystallographic Data Center and assigned the following deposition numbers: CCDC 1058838 and 1455648 for compounds **4u** and **5v**, respectively.

**Figure 3:**
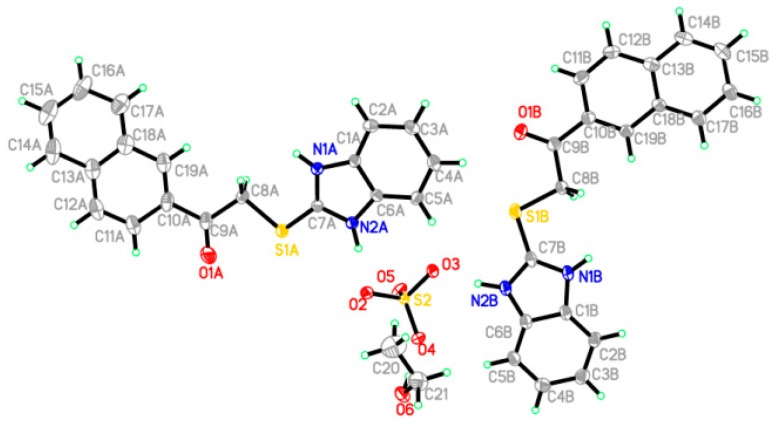
An ORTEP diagram of the final X-ray structure of compound **4u**.

**Figure 4:**
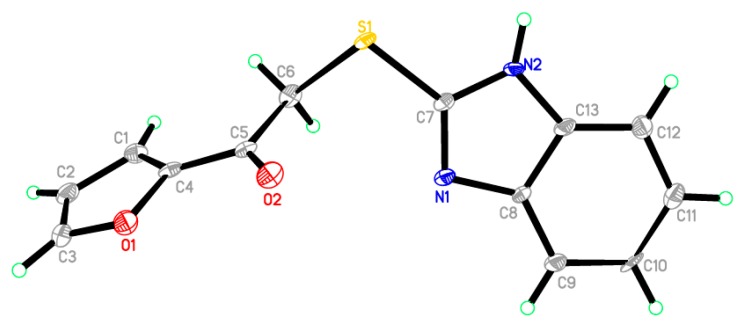
An ORTEP diagram of the final X-ray structure of compound **5v**.

In Figure 2 and Figure 3, the crystallographic structures of compounds **4u** and **5v** are represented, respectively. The exact structure is unambiguously defined by the single crystal X-ray study on both derivatives. The crystal structure of **4u** confirmed two crystallographically independent cation molecules with one sulfate anion, in the presence of one molecule of ethanol in its asymmetric unit, as shown in Figure 3. The asymmetric unit of **5v** contains one molecule only, as depicted in Figure 4. Table 1 listed the crystallographic data and the refinement for the crystals. Table 2 and Table 3 summarized some selected geometric parameters for **4u** and **5v**, respectively. Also, Figure S1 (in Supplementary Materials) displayed the molecular packing of compound **5v**, while Table S1 showed the hydrogen-bond geometry (Å, °) for compound **5v**.

### 2.3. Biological Evaluation of the Target Derivatives as Anti-Cancer Agents

#### 2.3.1. In Vitro Anti-Proliferative Activity against MDA-MB-468

The WST-1 assay, as described by Ngamwongsatit et al. [37], was adopted to evaluate the anti-proliferative activity of the synthesized 2-((benzimidazol-2-yl)thio)-1-arylethan-1-ones **5a**–**w** against human breast cancer cell line MDA-MB-468. 5-FU (florouracil) was selected as a positive control due to its broad spectrum of anticancer activity. The anti-proliferative activity was expressed as growth inhibitory concentration (IC_50_) values, which represent the compound concentrations required to produce a 50% inhibition of cell growth after 48 hours of incubation compared to untreated controls (Table 4).

The obtained results of the tested benzimidazole derivatives **5a**–**w** indicated that most of the prepared compounds showed good to moderate anti-proliferative activity against the tested MDA-MB-468 cancer cell line. Compound **5k** merged as the most potent member against MDA-MB-468 (IC_50_ = 19.90 ± 1.37 µM) as it was 2.1 times more potent and efficacious than 5-fluorouracil (IC_50_ = 41.26 ± 3.77 µM). Moreover, analogs **5a**, **5f**, and **5j**–**t** showed superior anti-proliferative activity (IC_50_ values ranging from 21.98 ± 1.91 to 26.60 ± 2.24 µM) compared to 5-fluorouracil, the reference drug, (IC_50_ = 41.26 ± 3.77 µM). In addition, compounds **5d**, **5i**, **5v**, and **5w** with IC_50_ = 31.97 ± 3.07, 32.80 ± 3.17, 30.34 ± 3.01 and 28.17 ± 2.24 µM, respectively, displayed good activity against MDA-MB-468. On the other hand, compounds **5b**, **5c**, **5g**, **5h**, and **5u** were moderately active against MDA-MB-468 with IC_50_ values ranging from 50.78 ± 5.11 to 74.25 ± 6.23 µM.

#### 2.3.2. Structure Activity Relationship Study (SAR Study) of the Target Compounds

Observing the results in Table 4, valuable data could be extracted regarding the structure activity correlation of our compounds. Foremost, the effect of grafting diverse substituents on the terminal aryl moiety on the activities of the synthesized compounds **5a**–**t** was closely investigated. Compound **5a** bearing unsubstituted phenyl group showed good activity (IC_50_ = 22.31 ± 2.04 µM) in comparison to 5-fluorouracil (IC_50_ = 41.26 ± 3.77 µM), implying a doubling of the anti-proliferative activity.

Introduction of fluorine atom, a classical bioisostere of the hydrogen atom, at the 4-position as in compound **5f** resulted in comparable activity to the unsubstituted analogue **5a** (IC_50_ = 24.96 ± 2.55 and 22.31 ± 2.04 µM, respectively). Interestingly, transferring the fluorine atom from the 4-position to the 2-position, **5e**, resulted in an inactive derivative. Again, di-substitution with two fluorine atoms in the 2- and 4-positions, **5g**, was not favorable to the activity (IC_50_ = 50.78 ± 5.11 µM) compared to the mono 4-F substituted derivative **5f** (IC_50_ = 24.96 ± 2.55 µM). Incorporation of more bulky halogens as chlorine and bromine led to compounds **5d** and **5h**, respectively, with decreased activity (IC_50_ = 31.97 ± 3.07 and 53.83 ± 6.53 µM, respectively), suggesting that incorporation of a small halogen fluorine atom only in the 4-position is markedly advantageous to the activity. The order of activities of the halogenated derivatives **5d**–**h** decreased in the order of 4-F > 4-Cl > 2,4-di-F > 4-Br > 2-F. Also, grafting an electron-withdrawing nitro group as in compound **5i** resulted only in moderate improvement of the activity (IC_50_ = 32.80 ± 3.17 µM) compared to 5-FU (IC_50_ = 41.26 ± 3.77 µM), while introduction of methyl or amino groups, electron-donating groups, reduced the activity against MDA-MB-468, as shown in **5b** and **5c** analogs (IC_50_ = 74.25 ± 6.23 and 53.79 ± 5.02 µM, respectively). Contrariwise, substitution with electron-donating hydroxyl, methoxy, or ethoxy groups as in compounds **5j**–**t** maintained the activity in the good range of activity regardless of their positions or numbers (IC_50_ = (19.90 ± 1.37)–(26.60 ± 2.24) µM).

On the other hand, scrutinizing the anti-proliferative activity of compounds **5u**–**w** gave us insight about the effect of exchanging the phenyl group of **5a** for other aryl or heteroaryl moieties. Replacement of the phenyl ring of **5a** with a naphthalin-2-yl group in compound **5u** decreased the activity (IC_50_ = 66.45 ± 7.12 µM). Moreover, bioisosteric replacement of the phenyl moiety with 2-furyl or 2-thienyl groups (compounds **5v** and **5w**) moderately reduced the activity (IC_50_ = 30.34 ± 3.01 and 28.17 ± 2.24 µM, respectively). In conclusion, we can assume that incorporation of an unsubstituted phenyl group or its substitution with electron-donating hydroxyl, methoxy, or ethoxy groups is beneficial for activity against the MDA-MB-468 cell line, while introduction of heterocycles, such as 2-furyl or 2-thienyl, could not effectively replace the phenyl ring.

#### 2.3.3. Cell-Cycle Analysis and Apoptotic Changes Investigation of Compound **5k**

Cell reproduction necessitates DNA replication with a concomitant nuclear division followed by cytoplasmic partitioning to sporulate two daughter cells. Such a successive routine is known as the “cell cycle” and involves four distinguishable phases. The G1 phase is a gap integrated between the M phase (nuclear division) and the S phase (DNA synthesis); another gap called G2 phase also occurs between S and M. These gaps permit the repair of DNA damage and replication errors [38].

**Figure 5:**
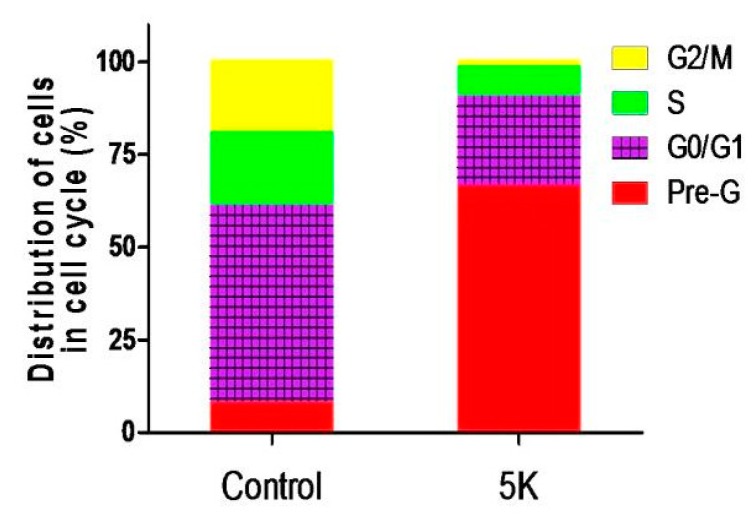
Bar chart shows percentage of MDA-MB-468 cells at each phase of cell cycle in control cells and cells treated with compound **5k**.

To understand the mechanism behind the tumor suppression activity of the prepared compounds, the most active member in this study, **5k**, was selected to be further investigated regarding its effects on cell cycle progression and its potential apoptotic effects in the MDA-MB-468 cell line. The MDA-MB-468 cells were treated with IC_50_ concentration of compound **5k** for 24 h and its effect on the normal cell cycle was detected by fluorescence-activated cell sorting (FACS) analysis (Figure 5, Figure S2). Interestingly, exposure of MDA-MB-468 cells to **5k** induced a remarkable augmentation in the proportion of cells at pre-G1 phase by 8.13-fold. The increase was accompanied by concomitant noteworthy mitigation in the percentage of cells at the G0/G1, S, and G2/M phases by 2.21-, 2.43-, and 11.83-fold in comparison to the control, respectively.

#### 2.3.4. Evaluation of the Apoptotic Effect of Compound **5k** by Fluorescein Isothiocyanate (FITC)-Labeled Annexin V (Annexin V–FITC) Assay

The apoptotic effect of **5k** was further evaluated by Annexin VFITC/PI (AV/PI) dual staining assay to examine the occurrence of phosphatidylserine externalization and also to understand whether it is due to physiological apoptosis or nonspecific necrosis.

**Figure 6:**
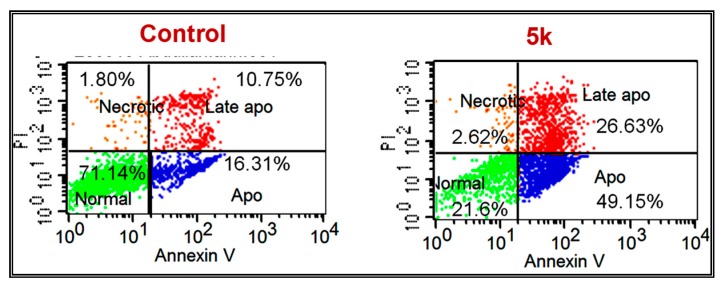
Fluorescein isothiocyanate (FITC)-labeled annexin V (Annexin V–FITC) staining. The cells were treated with dimethylsulfoxide (DMSO) as a control or with compound **5k** at IC_50_ concentration for 24 h. The experiment was done in triplicate.

**Figure 7:**
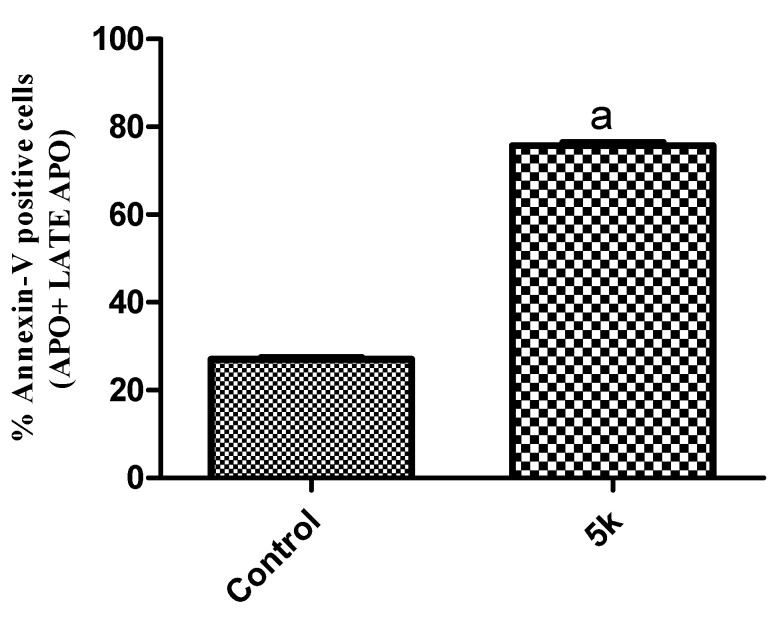
Effect of compound **5k** on the percentage of annexin V–FITC-positive staining in MDA-MB-468 cells. Data are presented as means ± S.D. ^a^ Indicates statistical difference from control at *p* < 0.0001.

In this study MDA-MB-468 cells were treated with compound **5k** for 48 h at 19.9 µM (IC_50_) to examine the apoptotic effect. It was observed that **5k** showed significant apoptosis against MDA-MB-468 cells, as shown in Figure 6 and Figure 7. Results indicated that **5k** showed 75.78% of apoptosis at 19.9 µM whereas 27.06% of apoptosis was observed in the control (untreated cells), comprising a 2.8-fold improvement compared to the control. This experiment suggests that **5k** significantly induces apoptosis in MDA-MB-468 cells.

### 2.4. 2 Dimensional-Quantitative Structure Activity Relationship (2D-QSAR) Analysis for the Anti-Proliferative Activity of the Prepared Derivatives ***5a***–***w***

#### 2.4.1. Elaboration of QSAR Model

QSAR analysis of the anti-tumor activity of the prepared derivatives **5a**–**w** was performed to establish a correlation between the biochemical data and the compound structures; moreover, it aids us in identifying the positive and negative structural features within the three scaffolds. DS 2.5 software (Discovery Studio 2.5, Accelrys, Co., Ltd., Accelrys, San Diego, CA, USA) was used to run the analysis.

A set of 21 synthesized derivatives (**5a**–**d**, **5f**–**I**, and **5k**–**w**) was applied as a training set with their experimentally detected logIC_50_ against the MDA-MB-468 cancer cell line in the QSAR modeling. The two remaining synthesized members were used as an external test set to assess the predictive power and validate the established QSAR model. Various molecular descriptors for the training set molecules were calculated using the “Calculate Molecular Properties” module. 2D Descriptors entangled: topological descriptors, molecular properties, molecular property counts, AlogP, surface area, and volume. As for the 3D descriptors: dipole, principal moments of inertia, jurs descriptors, surface area, and volume, and shadow indices. To search for the best QSAR regression equation, genetic function approximation (GFA) was utilized, i.e., multiple linear regression modeling (MLR).

#### 2.4.2. QSAR Study Results

The best performing QSAR model is represented by Equation (1);

Potency (LogIC_50_) against MDA-MB-468 cell line

LogIC_50_ = 4.1269 + 0.0599 Num_ExplicitAtoms − 0.0139 Molecular_SAVol + 3.0316 CHI_V_3_C − 0.0920 Jurs_RPCS (1)

**Figure 8:**
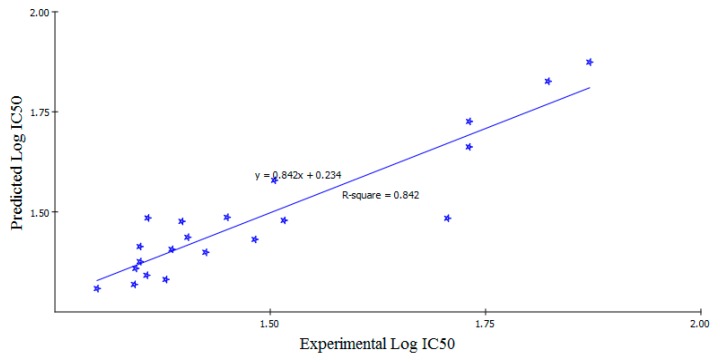
Predicted versus experimental logIC_50_ of the tested compounds against MDA-MB-468 cell line according to Equation (1) (*r*^2^ = 0.842).

Adopting Equation (1), QSAR model was graphically represented. This was accomplished by plotting the experimental values against the predicted bioactivity values logIC_50_ for the training set compounds, as shown in Figure 8. Also, the estimated and experimental activities data and the calculated descriptors of the training set compounds were summarized in Table 5. The Least-Squares method was used to build the models, *r*^2^ = 0.842, *r*^2^ (adj) = 0.803, *r*^2^ (pred) = 0.795, Least-Squared error = 0.004 for model 1, where *r*^2^ (adj) is *r*^2^ adjusted for the number of terms in the model; *r*^2^ (pred) is the prediction *r*^2^, equivalent to *q*^2^ from a leave-one-out cross-validation.

#### 2.4.3. QSAR Validation

Two of the prepared compounds (**5e** and **5j**) were utilized to carry out the external validation of the determined QSAR equation. **5e** and **5j** were chosen as they exhibit mild and excellent activities. The observed activities versus those provided by QSAR study are presented in Table 6.

### 2.5. Theoretical Kinetic ADME Study of the Target Derivatives **5a**–**w**

A theoretical kinetic study carried out by Discovery Studio 2.5 software (Accelrys) was adopted to predict the ADME of the prepared derivatives **5a**–**w**, Table 7. The lipophilicity was evaluated by calculating AlogP98, whereas the PSA_2D descriptor was adopted to estimate the polar surface area. Moreover, solubility level was predicted where all members of this study seemed to possess low solubility. In accordance with this anticipation, absorption levels implicate that they are well absorbed. Also, they are predicted to be non-inhibitors of CYP2D not to mention compounds **5b**, **5q**, and **5s**, which are expected to inhibit CYP2D. LogP for compound **5k** was determined experimentally and found to equal 3.75. It is worth mentioning that all compounds passed Lipinski’s rule of five.

## 3. Discussion

In summary, a novel series of 2-((benzimidazol-2-yl)thio)-1-arylethan-1-one derivatives **5a**–**w** has been synthesized**.** Their anti-proliferative activity against triple-negative breast cancer MDA-MB-468 cells was evaluated. Compound **5k** was found to be the most active compound in this study with IC_50_ value of 19.90 ± 1.37 µM as it was 2.1 times more potent and efficacious than 5-fluorouracil (IC_50_ = 41.26 ± 3.77 µM). Also, analogs **5a**, **5f**, and **5j**–**t** possessed excellent anti-proliferative activity with IC_50_ values ranging from 21.98 ± 1.91 to 26.60 ± 2.24 µM, which are better than the used reference drug. The preliminary SAR study showed that incorporation of unsubstituted phenyl group or its substitution with electron-donating hydroxyl, methoxy, or ethoxy groups are essential elements for the anti-tumor activity against MDA-MB-468, while introduction of heterocycles, such as 2-furyl or 2-thienyl, could not effectively replace the phenyl ring. In a cell-cycle analysis, compound **5k** increased the percentage of MDA-MB-468 cells at pre-G1 by 8.13-fold and G2/M phase by 11.83-fold. Furthermore, treatment of MDA-MB-468 cells with **5k** led to a marked increase in the percentage of annexin V–FITC-positive apoptotic cells (UR + LR) by 2.8-fold compared to the control. In addition, a QSAR model was established to investigate the structural requirements controlling activity against MDA-MB-468. Of note, the anticipated activities by the QSAR model were very near to the experimentally determined activities. Accordingly, this model could be conveniently applied for the prediction of more effective hits bearing the same structural framework. A theoretical kinetic study was constituted to anticipate the ADME of the prepared benzimidazoles. Moreover, single crystal X-ray diffraction has been included for compounds **4u** and **5v**.

Through this work we planned to add a scientific contribution for the treatment of the resistant type; TNBC by exploring different 2-benzimidazole derivatives. Our design was inspired by previously reported active scaffolds. Among the designed and synthetized molecules, many of them showed 2-fold increase in activity compared to 5-FU; this led us to develop a fruitful SAR analysis that will be a guideline for our future work.

## 4. Experimental

### 4.1. Chemistry

#### 4.1.1. General

Melting points were determined using a Gallenkamp melting point apparatus (WeissTechnik, Loughborough, UK) and are uncorrected. Infrared (IR) Spectra were recorded as KBr disks using the Perkin Elmer FT-IR Spectrum BX apparatus (PerkinElmer, Boston, MA, USA). Mass spectra were measured on an Agilent TripleQuadrupole 6410 QQQ LC/MS equipped with an ESI (electrospray ionization) source (Agilent Technologies, Santa Clara, CA, USA). NMR Spectra were recorded on a Bruker NMR spectrometer (Bruker, Karlsruhe, Germany). ^1^H spectrum was run at 500 MHz and ^13^C spectrum was run at 125 MHz in deuterated dimethyl sulfoxide (DMSO-*d*_6_). Chemical shifts are expressed in δ values (ppm) using the solvent peak as internal standard. All coupling constant (*J*) values are given in hertz. The abbreviations used are as follows: s, singlet; d, doublet; m, multiplet. Elemental analyses were carried out at the Regional Center for Mycology and Biotechnology, Al-Azhar University, Cairo, Egypt. Analytical thin layer chromatography (TLC) on silica gel plates containing UV indicator (Merck KGaA, Darmstadt, Germany) was employed routinely to follow the course of reactions and to check the purity of products. All reagents and solvents were purified and dried by standard techniques. Compounds **4** & **5a**, **b**, **f**, **h**, **4** & **5l**–**o**, **4** & **5q**, **r**, **t**, **v**, and **w**, are previously reported [28].

#### 4.1.2. Benzoimidazole-2-Thiol **2**

Prepared according to the reported procedures [39].

#### 4.1.3. General Procedures for Synthesis of Sulfate Salts **4a**–**w**

Prepared according to the reported procedures [28].

2-((2-(4-Aminophenyl)-2-oxoethyl)thio)-1*H*-benzo[d]imidazol-3-ium sulfate (**4c**). White crystals, (yield 97%), m.p. 210–213 °C; IR (KBr, *ν* cm^−1^): 3450 (NH) and 1684 (C=O); ^1^H-NMR (DMSO-*d*_6_) δ ppm: 5.23 (s, 2H, CH_2_), 6.65 (d, 2H, H-3 and H-5 of 4-NH_2_C_6_H_4_, *J* = 8.5 Hz), 6.70 (s, 2H, NH_2_), 7.49–7.51 (m, 2H, H-5 and H-6 of 2-mercaptobenzimidazole), 7.71–7.73 (m, 2H, H-4 and H-7 of 2-mercaptobenzimidazole), 7.87 (d, 2H, H-2 and H-6 of 4-NH_2_C_6_H_4_, *J* = 8.5 Hz), 10.55 (s, 1H, NH), 12.64 (s, 1H, NH); ^13^C-NMR (DMSO-*d*_6_) δ ppm: 41.55 (CH_2_), 113.40, 113.59, 114.66, 125.76, 130.92, 131.62, 132.74, 151.68, 189.11, 195.74 (C=O); ESI MS *m*/*z*: 665 [M + 1]^+^; Anal. Calcd. for C_30_H_28_N_6_O_6_S_3_: C, 54.20; H, 4.25; N, 12.64; Found C, 54.28; H, 4.21; N, 12.75.

2-((2-(4-Chlorophenyl)-2-oxoethyl)thio)-1*H*-benzo[d]imidazol-3-ium sulfate (**4d**). White crystals, (yield 98%), m.p. 227–230 °C; IR (KBr, *ν* cm^−1^): 3400 (NH) and 1683 (C=O); ^1^H-NMR (DMSO-*d*_6_) δ ppm: 5.29 (s, 2H, CH_2_), 7.37–7.40 (m, 2H, H-5 and H-6 of 2-mercaptobenzimidazole), 7.62–7.65 (m, 2H, H-4 and H-7 of 2-mercaptobenzimidazole), 7.68 (d, 2H, H-3 and H-5 of 4-ClC_6_H_4_, *J* = 8.5 Hz), 8.08 (d, 2H, H-2 and H-6 of 4-ClC_6_H_4_, *J* = 8.5 Hz), 10.55 (s, 1H, NH), 12.61 (s, 1H, NH); ^13^C-NMR (DMSO-*d*_6_) δ ppm: 56.50 (CH_2_), 113.84, 120.85, 122.77, 124.68, 129.53, 130.89, 134.12, 134.86, 139.48, 150.64, 192.07 (C=O); ESI MS *m*/*z*: 703 [M + 1]^+^, 704 [M + 2]^+^; Anal. Calcd. for C_30_H_24_Cl_2_N_4_O_6_S_3_: C, 15.21; H, 3.44; N, 7.96; Found C, 15.30; H, 3.49; N, 8.02.

2-((2-(2-Fluorophenyl)-2-oxoethyl)thio)-1*H*-benzo[d]imidazol-3-ium sulfate (**4e**). White crystals, (yield 97%), m.p. 219–222 °C; IR (KBr, *ν* cm^−1^): 3435 (NH) and 1669 (C=O); ^1^H-NMR (DMSO-*d*_6_) δ ppm: 5.15 (s, 2H, CH_2_), 7.37–7.40 (m, 2H, H-5 and H-6 of 2-mercaptobenzimidazole), 7.41–7.48 (m, 2H, Ar–H), 7.60–7.62 (m, 2H, H-4 and H-7 of 2-mercaptobenzimidazole), 7.75–7.79 (m, 1H, Ar–H), 7.94 (t, 1H, H-6 of 2-FC_6_H_4_, *J* = 7.5 Hz), 10.64 (s, 1H, NH), 12.73 (s, 1H, NH). ESI MS *m*/*z*: 671 [M + 1]^+^; Anal. Calcd. for C_30_H_24_F_2_N_4_O_6_S_3_: C, 53.72; H, 3.61; N, 8.35; Found C, 53.67; H, 3.65; N, 8.38.

2-((2-(2,4-Difluorophenyl)-2-oxoethyl)thio)-1*H*-benzo[d]imidazol-3-ium sulfate (**4g**). White crystals, (yield 96%), m.p. 206–209 °C; IR (KBr, *ν* cm^−1^): 3468 (NH) and 1680 (C=O); ^1^H-NMR (DMSO-*d*_6_) δ ppm: 5.27 (s, 2H, CH_2_), 7.30 (t, 1H, Ar–H, *J* = 8.5 Hz), 7.43–7.46 (m, 2H, H-5 and H-6 of 2-mercaptobenzimidazole), 7.52 (t, 1H, Ar–H, *J* = 9.0 Hz), 7.65–7.69 (m, 2H, H-4 and H-7 of 2-mercaptobenzimidazole), 8.03 (t, 1H, Ar–H, *J* = 9.0 Hz),10.52 (s, 1H, NH), 12.67 (s, 1H, NH); ^13^C-NMR (DMSO-*d*_6_) δ ppm: 44.06 (CH_2_), 105.70, 105.91, 106.12, 113.09, 113.27, 113.76, 120.81, 125.25, 133.50, 133.85, 150.71, 189.03 (C=O). ESI MS *m*/*z*: 707 [M + 1]^+^; Anal. Calcd. for C_30_H_22_F_4_N_4_O_6_S_3_: C, 50.99; H, 3.14; N, 7.93; Found C, 50.91; H, 3.18; N, 8.01.

2-((2-(2-Hydroxyphenyl)-2-oxoethyl)thio)-1*H*-benzo[d]imidazol-3-ium sulfate (**4j**). White crystals, (yield 98%), m.p. 238–240 °C; IR (KBr, *ν* cm^−1^): 3466 (NH) and 1684 (C=O); ^1^H-NMR (DMSO-*d*_6_) δ ppm: 5.30 (s, 2H, CH_2_), 7.02–7.11 (m, 4H, Ar–H), 7.38–7.42 (m, 2H, H-5 and H-6 of 2-mercaptobenzimidazole), 7.67–7.70 (m, 2H, H-4 and H-7 of 2-mercaptobenzimidazole), 9.59 (s, 1H, OH), 10.60 (s, 1H, NH), 12.87 (s, 1H, NH). ESI MS *m*/*z*: 667 [M + 1]^+^, 668 [M + 2]^+^; Anal. Calcd. for C_30_H_26_N_4_O_8_S_3_: C, 54.04; H, 3.93; N, 8.40; Found C, 54.12; H, 3.91; N, 8.46.

2-((2-(3-Hydroxyphenyl)-2-oxoethyl)thio)-1*H*-benzo[d]imidazol-3-ium sulfate (**4k**). White crystals, (yield 97%), m.p. 228–230 °C; IR (KBr, *ν* cm^−1^): 3418 (NH) and 1670 (C=O); ^1^H-NMR (DMSO-*d*_6_) δ ppm: 5.34 (s, 2H, CH_2_), 7.11 (d, 1H, H-4 of 3-OHC_6_H_4_, *J* = 8.0 Hz), 7.40–7.44 (m, 2H, H-2 and H-3 of 3-OHC_6_H_4_), 7.47–7.49 (m, 2H, H-5 and H-6 of 2-mercaptobenzimidazole), 7.54 (d, 1H, H-6 of 3-OHC_6_H_4_, *J* = 8.0 Hz), 7.70–7.72 (m, 2H, H-4 and H-7 of 2-mercaptobenzimidazole), 9.73 (s, 1H, OH), 10.48 (s, 1H, NH), 12.61 (s, 1H, NH); ^13^C-NMR (DMSO-*d*_6_) δ ppm: 41.58 (CH_2_), 113.69, 115.09, 120.03, 121.82, 125.59, 130.55, 133.19, 136.46, 151.08, 158.23, 192.52 (C=O). ESI MS *m*/*z*: 667 [M + 1]^+^; Anal. Calcd. for C_30_H_26_N_4_O_8_S_3_: C, 54.04; H, 3.93; N, 8.40; Found C, 54.13; H, 4.03; N, 8.46.

2-((2-(2,4-Dimethoxyphenyl)-2-oxoethyl)thio)-1*H*-benzo[d]imidazol-3-ium sulfate (**4p**). White crystals, (yield 98%), m.p. 235–237 °C; IR (KBr, *ν* cm^−1^): 3377 (NH) and 1680 (C=O); ^1^H-NMR (DMSO-*d*_6_) δ ppm: 3.88 (s, 3H, OCH_3_), 4.00 (s, 3H, OCH_3_), 5.08 (s, 2H, CH_2_), 6.67 (d, 1H, H-5 of 2,4-(OCH_3_)_2_C_6_H_3_, *J* = 9.0 Hz), 6.74 (s, 1H, H-3 of 2,4-(OCH_3_)_2_C_6_H_3_), 7.43–7.44 (m, 2H, H-5 and H-6 of 2-mercaptobenzimidazole), 7.65–7.66 (m, 2H, H-4 and H-7 of 2-mercaptobenzimidazole), 7.79 (d, 1H, H-6 of 2,4-(OCH_3_)_2_C_6_H_3_, *J* = 9.0 Hz), 10.46 (s, 1H, NH), 12.37 (s, 1H, NH); ^13^C-NMR (DMSO-*d*_6_) δ ppm: 45.31 (CH_2_), 56.33 (OCH_3_), 56.73 (OCH_3_), 98.92, 107.24, 113.37, 117.65, 121.38, 125.12, 133.06, 151.57, 162.02, 165.95, 191.78 (C=O). ESI MS *m*/*z*: 755 [M + 1]^+^; Anal. Calcd. for C_34_H_34_N_4_O_10_S_3_: C, 54.10; H, 4.54; N, 7.42; Found C, 54.16; H, 4.59; N, 7.38.

2-((2-(3,4,5-Trimethoxyphenyl)-2-oxoethyl)thio)-1*H*-benzo[d]imidazol-3-ium sulfate (**4s**). White crystals, (yield 98%), m.p. 213–215 °C; IR (KBr, *ν* cm^−1^): 3427 (NH) and 1675 (C=O); ^1^H-NMR (DMSO-*d*_6_) δ ppm: 3.77 (s, 6H, 2OCH_3_), 3.89 (s, 12H, 4OCH_3_), 5.29 (s, 4H, 2CH_2_), 7.40–7.67 (m, 12H, Ar–H), 10.67 (s, 2H, 2NH), 12.49 (s, 2H, 2NH). ESI MS *m*/*z*: 815 [M + 1]^+^, 816 [M + 2]^+^; Anal. Calcd. for C_36_H_38_N_4_O_12_S_3_: C, 53.06; H, 4.70; N, 6.88; Found C, 52.96; H, 4.63; N, 6.80.

2-((2-(Naphthalen-2-yl)-2-oxoethyl)thio)-1*H*-benzo[d]imidazol-3-ium sulfate (**4u**). White crystals, (yield 96%), m.p. 246–250 °C; IR (KBr, *ν* cm^−1^): 3412 (NH) and 1670 (C=O); ^1^H-NMR (DMSO-*d*_6_) δ ppm: 5.38 (s, 2H, CH_2_), 7.31–7.33 (m, 2H, H-5 and H-6 of 2-mercaptobenzimidazole), 7.57–7.60 (m, 2H, H-4 and H-7 of 2-mercaptobenzimidazole), 7.66–7.74 (m, 2H, H-6 and H-7 of naphthalene), 8.04 (d, 2H, Ar–H, *J* = 8.5 Hz), 8.08 (d, 1H, Ar–H, *J* = 8.5 Hz), 8.17 (d, 1H, Ar–H, *J* = 8.0 Hz), 8.85 (s, 1H, H-1 of naphthalene), 10.54 (s, 1H, NH), 12.68 (s, 1H, NH); ^13^C-NMR (DMSO-*d*_6_) δ ppm: 56.49 (CH_2_), 113.96, 123.93, 124.13, 127.72, 128.26, 129.01, 130.14, 131.27, 132.56, 132.83, 135.79, 136.26, 150.58, 193.14 (C=O). ESI MS *m*/*z*: 735 [M + 1]^+^; Anal. Calcd. for C_38_H_30_N_4_O_6_S_3_: C, 62.11; H, 4.11; N, 7.62; Found C, 62.18; H, 4.13; N, 7.57.

#### 4.1.4. General Procedure for Preparation of the Target Derivatives **5a**–**w**

An aqueous solution (10 mL) of sodium bicarbonate was added to a stirred suspension of the adequate sulfate salts **4a**–**w** (4 mmol) in water (20 mL). The mixture was stirred for 2 h at room temperature. The obtained solid was collected by filtration, washed several times with water, then dried and recrystallized from ethanol to furnish compounds **5a**–**w**.

2-((1H-Benzo[d]imidazol-2-yl)thio)-1-(4-aminophenyl)ethan-1-one (**5c**). White crystals (yield 85%), m.p. 175–178 °C; IR (KBr, *ν* cm^−1^): 3412 (NH) and 1680 (C=O); ^1^H-NMR (DMSO-*d*_6_) δ ppm: 4.87 (s, 2H, CH_2_), 6.21 (s, 2H, NH_2_), 6.59 (d, 2H, H-3 and H-5 of 4-NH_2_C_6_H_4_, *J* = 9.0 Hz), 7.10–7.14 (m, 4H, H-4, H-5, H-6 and H-7 of 2-mercaptobenzimidazole), 7.76 (d, 2H, H-2 and H-6 of 4-NH_2_C_6_H_4_, *J* = 8.5 Hz), 12.53 (s, 1H, NH); ^13^C-NMR (DMSO-*d*_6_) δ ppm: 40.57 (CH_2_), 109.93, 113.03, 121.82, 122.78, 123.27, 131.42, 132.70, 150.41, 154.72, 190.66 (C=O); ESI MS *m*/*z*: 284 [M + 1]^+^; Anal. Calcd. for C_15_H_13_N_3_OS: C, 63.58; H, 4.62; N, 14.83; Found C, 63.81; H, 4.60; N, 14.89.

2-((1H-Benzo[d]imidazol-2-yl)thio)-1-(4-chlorophenyl)ethan-1-one (**5d**). White crystals (yield 90%), m.p. 179–181 °C (reported: 189–191 °C [40]); IR (KBr, *ν* cm^−1^): 3410 (NH) and 1675 (C=O); ^1^H-NMR (DMSO-*d*_6_) δ ppm: 5.04 (s, 2H, CH_2_), 7.09–7.13 (m, 2H, H-5 and H-6 of 2-mercaptobenzimidazole), 7.40–7.42 (m, 2H, H-4 and H-7 of 2-mercaptobenzimidazole), 7.64 (d, 2H, H-3 and H-5 of 4-ClC_6_H_4_, *J* = 8.0 Hz), 8.08 (d, 2H, H-2 and H-6 of 4-ClC_6_H_4_, *J* = 8.5 Hz), 12.64 (s, 1H, NH); ^13^C-NMR (DMSO-*d*_6_) δ ppm: 40.49 (CH_2_), 112.45, 121.87, 129.41, 130.82, 134.67, 139.06, 149.82, 193.09 (C=O); ESI MS *m*/*z*: 302.9 [M]^+^, 304.9 [M + 2]^+^.

2-((1H-Benzo[d]imidazol-2-yl)thio)-1-(2-fluorophenyl)ethan-1-one (**5e**). White crystals (yield 89%), m.p. 145–148 °C; IR (KBr, *ν* cm^−1^): 3420 (NH) and 1654 (C=O); ^1^H-NMR (DMSO-*d*_6_) δ ppm: 4.93 (s, 2H, CH_2_), 7.09–7.12 (m, 2H, H-5 and H-6 of 2-mercaptobenzimidazole), 7.37–7.49 (m, 4H, Ar–H), 7.70–7.74 (m, 1H, Ar–H), 8.08 (t, 1H, Ar–H, *J* = 7.5 Hz), 12.66 (s, 1H, NH); ^13^C-NMR (DMSO-*d*_6_) δ ppm: 43.11 (CH_2_), 109.23, 117.29, 118.50, 121.69, 122.18, 124.61, 125.38, 131.09, 136.01, 149.80, 157.31, 160.50, 162.52, 191.90 (C=O); ESI MS *m*/*z*: 287 [M + 1]^+^, 288 [M + 2]^+^; Anal. Calcd. for C_15_H_11_FN_2_OS: C, 62.92; H, 3.87; N, 9.78; Found C, 63.09; H, 3.90; N, 9.84.

2-((1H-Benzo[d]imidazol-2-yl)thio)-1-(2,4-difluorophenyl)ethan-1-one (**5g**). White crystals (yield 94%), m.p. 116–120 °C; IR (KBr, *ν* cm^−1^): 3420 (NH) and 1675 (C=O); ^1^H-NMR (DMSO-*d*_6_) δ ppm: 4.91 (s, 2H, CH_2_), 7.08–7.12 (m, 2H, H-5 and H-6 of 2-mercaptobenzimidazole), 7.26 (t, 1H, Ar–H, *J* = 8.5 Hz), 7.39–7.41 (m, 2H, H-4 and H-7 of 2-mercaptobenzimidazole), 7.47 (t, 1H, Ar–H, *J* = 9 Hz), 8.00 (q, 1H, Ar–H, *J* = 8.5 Hz), 12.61 (s, 1H, NH); ^13^C-NMR (DMSO-*d*_6_) δ ppm: 42.95 (CH_2_), 105.54, 105.75, 105.96, 112.89, 113.04, 121.88, 133.35, 149.73, 190.71 (C=O); ESI MS *m*/*z*: 304 [M]^+^, 305 [M + 1]^+^; Anal. Calcd. for C_15_H_10_F_2_N_2_OS: C, 59.20; H, 3.31; N, 9.21; Found C, 59.46; H, 3.29; N, 9.32.

2-((1H-Benzo[d]imidazol-2-yl)thio)-1-(2-hydroxyphenyl)ethan-1-one (**5j**). White crystals (yield 85%), m.p. 205–208 °C (reported: 201 °C [41]); IR (KBr, *ν* cm^−1^): 3408 (NH) and 1670 (C=O); ^1^H-NMR (DMSO-*d*_6_) δ ppm: 4.91 (s, 2H, CH_2_), 6.99–7.15 (m, 4H, Ar–H), 7.38–7.41 (m, 2H, H-4 and H-7 of 2-mercaptobenzimidazole), 7.83–7.95 (m, 2H, Ar–H), 9.37 (s, 1H, OH), 12.53 (s, 1H, NH); ESI MS *m*/*z*: 285 [M + 1]^+^.

2-((1H-Benzo[d]imidazol-2-yl)thio)-1-(3-hydroxyphenyl)ethan-1-one (**5k**). White crystals (yield 87%), m.p. 228–230 °C (reported: 224–227 °C [42]); IR (KBr, *ν* cm^−1^): 3336 (NH) and 1660 (C=O); ^1^H-NMR (DMSO-*d*_6_) δ ppm: 5.01 (s, 2H, CH_2_), 7.08–7.10 (m, 3H, H-5, H-6 of 2-mercaptobenzimidazole and H-4 of 3-OHC_6_H_4_), 7.37–7.45 (m, 4H, H-4, H-7 of 2-mercaptobenzimidazole and H-5, H-6 of 3-OHC_6_H_4_), 7.54 (s, 1H, H-2 of 3-OHC_6_H_4_), 9.89 (s, 1H, OH), 12.61 (s, 1H, NH); ^13^C-NMR (DMSO-*d*_6_) δ ppm: 40.47 (CH_2_), 114.96, 119.86, 121.28, 121.61, 130.43, 137.23, 150.01, 158.14, 193.72 (C=O); ESI MS *m*/*z*: 285.

2-((1H-Benzo[d]imidazol-2-yl)thio)-1-(2,4-dimethoxyphenyl)ethan-1-one (**5p**). White crystals (yield 92%), m.p. 208–211 °C; IR (KBr, *ν* cm^−1^): 3413 (NH) and 1655 (C=O); ^1^H-NMR (DMSO-*d*_6_) δ ppm: 3.92 (s, 3H, OCH_3_), 3.96 (s, 3H, OCH_3_), 4.80 (s, 2H, CH_2_), 6.65 (d, 1H, H-5 of 2,4-(OCH_3_)_2_C_6_H_3_, *J* = 9.0 Hz), 6.71 (s, 1H, H-3 of 2,4-(OCH_3_)_2_C_6_H_3_), 7.06–7.09 (m, 2H, H-5 and H-6 of 2-mercaptobenzimidazole), 7.40–7.41 (m, 2H, H-4 and H-7 of 2-mercaptobenzimidazole), 7.73 (d, 1H, H-6 of 2,4-(OCH_3_)_2_C_6_H_3_, *J* = 8.5 Hz), 12.60 (s, 1H, NH); ^13^C-NMR (DMSO-*d*_6_) δ ppm: 43.97 (CH_2_), 56.20 (OCH_3_), 56.60 (OCH_3_), 98.88, 104.25, 106.96, 110.65, 114.19, 118.27, 121.63, 132.88, 150.15, 161.52, 165.36, 193.07 (C=O); ESI MS *m*/*z*: 329 [M + 1]^+^; Anal. Calcd. for C_17_H_16_N_2_O_3_S: C, 62.18; H, 4.91; N, 8.53; Found C, 62.40; H, 4.94; N, 8.45.

2-((1H-Benzo[d]imidazol-2-yl)thio)-1-(3,4,5-trimethoxyphenyl)ethan-1-one (**5s**). White crystals (yield 88%), m.p. 233–235 °C; IR (KBr, *ν* cm^−1^): 3405 (NH) and 1670 (C=O); ^1^H-NMR (DMSO-*d*_6_) δ ppm: 3.77 (s, 3H, OCH_3_), 3.86 (s, 6H, OCH_3_), 5.04 (s, 2H, CH_2_), 7.12–7.43 (m, 6H, Ar–H), 12.68 (s, 1H, NH); ^13^C-NMR (DMSO-*d*_6_) δ ppm: 39.37 (CH_2_), 56.60 (OCH_3_), 60.67 (OCH_3_), 106.58, 121.92, 131.12, 142.73, 149.87, 153.29, 192.94 (C=O); Anal. Calcd. for C_18_H_18_N_2_O_4_S: C, 60.32; H, 5.06; N, 7.82; Found C, 60.41; H, 5.03; N, 7.75.

2-((1H-Benzo[d]imidazol-2-yl)thio)-1-(naphthalen-2-yl)ethan-1-one (**5u**). White crystals (yield 92%), m.p. 160–162 °C; IR (KBr, *ν* cm^−1^): 3415 (NH) and 1673 (C=O); ^1^H-NMR (DMSO-*d*_6_) δ ppm: 5.20 (s, 2H, CH_2_), 7.10–7.13 (m, 2H, H-5 and H-6 of 2-mercaptobenzimidazole), 7.41–7.43 (m, 2H, H-4 and H-7 of 2-mercaptobenzimidazole), 7.64–7.72 (m, 2H, Ar–H), 8.02–8.07 (m, 3H, Ar–H), 8.15 (d, 1H, Ar–H, *J* = 8.0 Hz), 8.85 (s, 1H, H-1 of naphthalene), 12.80 (s, 1H, NH); ^13^C-NMR (DMSO-*d*_6_) δ ppm: 40.42 (CH_2_), 121.92, 124.19, 127.59, 128.20, 128.91, 129.42, 130.12, 131.11, 132.59, 133.19, 135.69, 149.99, 193.90 (C=O); ESI MS *m*/*z*: 319 [M + 1]^+^; Anal. Calcd. for C_19_H_14_N_2_OS: C, 71.68; H, 4.43; N, 8.80; Found C, 71.81; H, 4.40; N, 8.73.

### 4.2. Biological Evaluation

#### 4.2.1. In Vitro Evaluation of the Anti-Proliferative Activity

The synthesized derivatives **5a**–**w** was evaluated for their anti-proliferative activity via the Stem Cell Therapy and Tissue Reengineering Program in the King Faisal Specialized Hospital and Research Center, Riyadh, Saudi Arabia. In vitro anti-proliferative activity was measured by the cell growth inhibition assay. This assay was conducted using a WST-1 reagent (Sigma-Aldrich Chemie Gmbh, Munich, Germany) for determination of the IC_50_ for each compound and the results are given in Table 4. MDA-MB-468 breast cancer cell line was purchased from the American Type Culture Collection (Manassas, Virginia, USA). Cells were maintained in Dulbecco’s modified Eagle’s medium (DMEM) (Sigma-Aldrich Chemie Gmbh), supplemented with 10% FBS (Lonza, Visp, Switzerland), 100 IU/mL penicillin, 100 mg/mL streptomycin, and 2 mmol/L l-glutamine (Sigma). Cells were seeded into 96-well plates at 0.4 × 10^4^/well and incubated overnight. The medium was replaced with a fresh one containing the desired concentrations of the test compounds. After 48 h, 10 µL of the WST-1 reagent were added to each well and the plates were re-incubated for 4 h at 37 °C. The amount of formazan was quantified using an ELISA reader (Thermo Fisher Scientific, Waltham, MA, USA) at 450 nm. The IC_50_ values were calculated according to the equation for Boltzmann sigmoidal concentration response curve using the nonlinear regression models (GraphPad, Prism Version 5, San Diego, CA, USA). The results reported are means of at least three separate experiments. Significant differences were analyzed by one-way analysis of variance (ANOVA) wherein the differences were considered to be significant at *p* < 0.05.

#### 4.2.2. Cell Cycle Analysis

The MDA-MB-468 cells were subjected to treatment with 19.90 µM of compound **5k** for 24 h. Consequently, the cells were washed twice with ice-cold phosphate buffered saline (PBS). The treated cells were collected by centrifugation, fixed in ice-cold 70% (*v*/*v*) ethanol, washed with PBS, re-suspended with 0.1 mg/mL RNase, stained with 40 mg/mL PI, and analyzed by flow cytometry using FACScalibur (Becton Dickinson, BD, San Jose, CA, USA). The cell cycle distributions were calculated using CellQuest software (Becton Dickinson).

#### 4.2.3. Annexin V–FITC Apoptosis Assay

The MDA-MB-468 cells were seeded as described above and then incubated with 19.90 µM of compound **5k** for 24 h. Cells were harvested, washed twice with PBS, and centrifuged. In brief, 10^5^ of cells were treated with annexin V–FITC and propidium iodide (PI) using the apoptosis detection kit (BD Biosciences, San Jose, CA, USA) according to the manufacturer’s protocol. Annexin V–FITC and PI binding were analyzed by flow cytometry on FACScalibur (BD Biosciences) without gating restrictions using 10,000 cells. Data were collected using logarithmic amplification of both the FL1 (FITC) and the FL2 (PI) channels. Quadrant analysis of co-ordinate dot plots was performed with CellQuest software. Unstained cells were used to adjust the photomultiplier voltage and for compensation setting adjustment to eliminate spectral overlap between the FL1 and the FL2 signal.

## Figures and Tables

**Table 1:** Crystallographic data and refinements for compounds **4u** and **5v**.

Compound	4u	5v
Crystal data		
Chemical formula	2(C_19_H_15_N_2_OS) C_2_H_6_O·SO_4_	C_13_H_10_N_2_O_2_S
*M*r	780.94	258.29
Crystal system, space group	Orthorhombic, *P*2_1_2_1_2_1_	Monoclinic, *P*2_1_/*c*
Temperature (*K*)	100	100
*a*, *b*, *c* (Å)	14.2409 (8), 15.8339 (9), 16.2043 (8)	11.4097 (7), 10.8336 (7), 10.1256 (7)
β (°)	90.00	114.165 (2)
*V* (Å^3^)	3653.9 (3)	1141.93 (13)
*Z*	4	4
Radiation type	Mo *Kα*	Mo *Kα*
µ (mm^−1^)	0.26	0.28
Crystal size (mm)	0.44 × 0.26 × 0.16	0.47 × 0.36 × 0.11
Data collection		
Diffractometer	CCD area detector diffractometer	Bruker APEX-II D8 venture diffractometer
Absorption correction	multi-scan, SADABS	multi-scan, SADABS
*T*_min_, *T*_max_	0.90, 0.92	0.89, 0.93
Number of measured, independent and observed [I > 2σ(I)] reflections	11,220, 11,220, 8647	9364, 1999, 1689
*R*_int_		0.090
Refinement		
R[*F*^2^ > 2ó(*F*^2^)], wR(*F*^2^), S	0.050, 0.121, 1.05	0.086, 0.250, 1.10
Number of reflections	11,220	1999
Number of parameters	494	163
Number of restraints	0	0
H-atom treatment	by a mixture of independent and constrained refinement	by a mixture of independent and constrained refinement
Δρ_max_, Δρ_min_ (e·Å^−3^)	0.76, −0.37	1.12, −0.87
CCDC number	1,058,838	1,455,648

**Table 2:** Selected geometric parameters (Å, °) for compound **4u**.

**Bond Distances**			
S1A–C7A	1.722 (3)	O6–C21	1.437 (6)
S1A–C8A	1.811 (3)	N1A–C7A	1.333 (4)
S1B–C7B	1.724 (3)	N1A–C1A	1.406 (4)
S1B–C8B	1.804 (3)	N2A–C6A	1.387 (4)
S2–O3	1.476 (3)	N2A–C7A	1.338 (4)
S2–O5	1.479 (3)	N1B–C7B	1.342 (4)
S2–O2	1.478 (3)	N1B–C1B	1.398 (4)
S2–O4	1.467 (2)	N2B–C7B	1.339 (4)
O1A–C9A	1.203 (6)	N2B–C6B	1.381 (4)
**Bond Angles**			
C7A–S1A–C8A	102.76 (15)	N1A–C7A–N2A	109.8 (3)
C7B–S1B–C8B	99.02 (15)	S1A–C7A–N1A	130.1 (2)
O3–S2–O5	108.55 (14)	S1A–C8A–C9A	105.5 (2)
O4–S2–O5	111.31 (14)	O1A–C9A–C10A	121.0 (4)
O3–S2–O4	110.49 (14)	O1A–C9A–C8A	120.6 (3)
O2–S2–O3	108.54 (16)	N1B–C1B–C2B	131.1 (3)
O2–S2–O4	109.46 (15)	N1B–C1B–C6B	106.5 (3)
O2–S2–O5	108.43 (14)	N2B–C6B–C5B	131.8 (3)
C1A–N1A–C7A	108.2 (3)	N2B–C6B–C1B	107.0 (3)
C6A–N2A–C7A	108.7 (3)	S1B–C7B–N1B	128.7 (3)
C1B–N1B–C7B	108.0 (3)	S1B–C7B–N2B	121.3 (2)
C6B–N2B–C7B	108.7 (3)	N1B–C7B–N2B	109.9 (3)
N1A–C1A–C2A	130.9 (3)	S1B–C8B–C9B	107.8 (2)
N1A–C1A–C6A	106.5 (3)	O1B–C9B–C10B	122.0 (3)
N2A–C6A–C1A	106.9 (3)	O1B–C9B–C8B	120.4 (3)
N2A–C6A–C5A	131.1 (3)	O6–C21–C20	111.9 (4)

**Table 3:** Selected geometric parameters (Å, °) for compound **5v**.

**Bond Distances**			
Cl1–C13	1.7361 (12)	N1–C1	1.3831 (13)
S1–C7	1.7521 (10)	N1–C7	1.3686 (12)
S1–C8	1.7949 (10)	N2–C6	1.3947 (13)
S1–C8	1.7949 (10)	N2–C6	1.3947 (13)
O1–C9	1.2146 (12)	N2–C7	1.3157 (12)
**Bond Angles**			
C6–S1–C7	102.2 (2)	S1–C6–C5	113.5 (4)
C3–O1–C4	106.5 (4)	S1–C7–N2	117.3 (3)
C7–N1–C8	103.8 (4)	N1–C7–N2	114.4 (5)
C7–N2–C13	106.7 (4)	S1–C7–N1	128.4 (4)
O1–C3–C2	111.2 (5)	N1–C8–C9	130.2 (4)
O1–C4–C5	116.2 (4)	N1–C8–C13	109.6 (4)
O1–C4–C1	109.8 (4)	N2–C13–C8	105.5 (5)
O2–C5–C6	123.4 (5)	N2–C13–C12	132.2 (5)

**Table 4:** In vitro anti-proliferative activity of compounds **5a**–**w** against breast MDA-MB-468 cancer cell line.

Compound	Aryl	IC_50_ (µM) ^a^
**5a**	C_6_H_5_	22.31 ± 2.04
**5b**	4-Me–C_6_H_4_	74.25 ± 6.23
**5c**	4-NH_2_–C_6_H_4_	53.79 ± 5.02
**5d**	4-Cl–C_6_H_4_	31.97 ± 3.07
**5e**	2-F–C_6_H_4_	NA ^b^
**5f**	4-F–C_6_H_4_	24.96 ± 2.55
**5g**	2,4-di-F–C_6_H_3_	50.78 ± 5.11
**5h**	4-Br–C_6_H_4_	53.83 ± 6.53
**5i**	4-NO_2_–C_6_H_4_	32.80 ± 3.17
**5j**	2-OH–C_6_H_4_	22.41 ± 2.13
**5k**	3-OH–C_6_H_4_	19.90 ± 1.37
**5l**	4-OH–C_6_H_4_	21.98 ± 1.91
**5m**	2-OCH_3_–C_6_H_4_	22.32 ± 2.06
**5n**	4-OCH_3_–C_6_H_4_	25.35 ± 2.15
**5o**	3-OH-4-OCH_3_–C_6_H_3_	22.06 ± 1.93
**5p**	2,4-(OCH_3_)_2_–C_6_H_3_	26.60 ± 2.24
**5q**	3,4-(OCH_3_)_2_–C_6_H_3_	24.30 ± 2.13
**5r**	3,4-O-CH_2_–*O*–C_6_H_3_	22.79 ± 1.87
**5s**	3,4,5-(OCH_3_)_3_–C_6_H_2_	22.72 ± 2.03
**5t**	4-OC_2_H_5_–C_6_H_4_	23.91 ± 2.19
**5u**	naphthalin-2-yl	66.45 ± 7.12
**5v**	furan-2-yl	30.34 ± 3.01
**5w**	thiophen-2-yl	28.17 ± 2.24
**5-Fluorouracil**		41.26 ± 3.77

^a^ IC_50_ values are the mean ± S.D. of three separate experiments; ^b^ NA: Compounds having IC_50_ value > 100 µM.

**Table 5:** Estimated IC_50_ data of the training set against MDA-MB-468 cell line and calculated descriptors governing IC_50_ according to Equation (1).

Compound	Experimental Activity (LogIC_50_)	Predicted Activity (LogIC_50_)	Residual	Num_ExplicitAtoms	Molecular_SAVol	CHI_V_3_C	Jurs_RPCS
**5a**	1.3485	1.4134	−0.0649	31	414.53	0.429	0.817
**5b**	1.8707	1.8742	−0.0035	34	430.44	0.596	0.834
**5c**	1.7307	1.6624	0.0683	33	426.68	0.525	0.736
**5d**	1.5047	1.5789	−0.0742	31	443.37	0.618	0.860
**5f**	1.3972	1.4763	−0.0791	31	423.74	0.492	0.807
**5g**	1.7057	1.4841	0.2216	31	432.96	0.536	0.752
**5h**	1.7310	1.7260	0.0050	31	462.96	0.756	0.844
**5i**	1.5159	1.4788	0.0371	33	441.92	0.541	0.928
**5k**	1.2989	1.3084	−0.0095	32	423.66	0.504	3.675
**5l**	1.3420	1.3186	0.0234	32	423.66	0.504	3.563
**5m**	1.3487	1.3758	−0.0271	35	445.34	0.477	0.720
**5n**	1.4040	1.4366	−0.0326	35	445.34	0.497	0.727
**5o**	1.3436	1.3587	−0.0151	36	454.48	0.553	2.662
**5p**	1.4249	1.3990	0.0259	39	476.16	0.545	0.630
**5q**	1.3856	1.4065	−0.0209	39	476.16	0.547	0.616
**5r**	1.3577	1.4848	−0.1271	34	444.90	0.528	0.633
**5s**	1.3564	1.3416	0.0148	43	506.98	0.579	0.296
**5t**	1.3786	1.3311	0.0475	38	465.86	0.497	0.710
**5u**	1.8225	1.8264	−0.0039	38	452.50	0.628	1.671
**5v**	1.4820	1.4312	0.0508	28	396.29	0.405	0.637
**5w**	1.4498	1.4865	−0.0367	28	417.77	0.523	0.655

**Table 6:** External validation of the established QSAR model.

Compound	Experimental Activity (LogIC_50_)	Predicted Activity (LogIC_50_)	Residual	Num_ExplicitAtoms	Molecular_SAVol	CHI_V_3_C	Jurs_RPCS
**5e**	2.1497	2.1497	0.00	31	423.74	0.473	0.795
**5j**	1.3504	1.3504	0.00	32	423.66	0.482	3.313

**Table 7:** Computer-aided ADME study of the prepared derivatives.

Compound	Alog*P*98 ^a^	PSA_2D ^b^	Solubility ^c^	Solubility Level ^d^	Absorption Level ^e^	CYP2D6 ^f^	CYP2D6 Probability ^g^
**5a**	3.684	43.616	−4.830	2	0	0	0.316
**5b**	4.171	43.616	−5.324	2	0	1	0.554
**5c**	2.938	70.156	−4.307	2	0	0	0.376
**5d**	4.349	43.616	−5.560	2	0	0	0.376
**5e**	3.890	43.616	−5.158	2	0	0	0.336
**5f**	3.890	43.616	−5.152	2	0	0	0.376
**5g**	4.095	43.616	−5.478	2	0	0	0.326
**5h**	4.433	43.616	−5.636	2	0	0	0.287
**5i**	3.579	86.440	−5.034	2	0	0	0.257
**5j**	3.442	64.432	−4.355	2	0	0	0.326
**5k**	3.442	64.432	−4.363	2	0	0	0.336
**5l**	3.442	64.432	−4.366	2	0	0	0.386
**5m**	3.668	52.547	−4.911	2	0	0	0.376
**5n**	3.668	52.547	−4.887	2	0	0	0.396
**5o**	3.426	73.362	−4.486	2	0	0	0.425
**5p**	3.652	61.477	−4.959	2	0	0	0.306
**5q**	3.652	61.477	−4.949	2	0	1	0.524
**5r**	3.453	61.477	−5.112	2	0	0	0.346
**5s**	3.635	70.407	−5.013	2	0	1	0.554
**5t**	4.017	52.547	−5.102	2	0	0	0.366
**5u**	3.287	28.624	−4.870	2	0	0	0.306
**5v**	3.080	56.171	−4.348	2	0	0	0.099
**5w**	3.410	43.616	−4.643	2	0	0	0.425

^a^ Lipophilicity descriptor; ^b^ Polar surface area; ^c^ Solubility parameter. (0–−2 = optimal, −2–−4 = good, −4–−6 = low, −6–−8 = very low); ^d^ Solubility level. (0 = extremely low, 1 = very low but possible, 2 = low, 3 = good, 4 = optimal); ^e^ Absorption level. (0 = good, 1 = moderate, 2 = low, 3 = very low); ^f^ CYP2D inhibition. (0 = non inhibitor, 1 = inhibitor); ^g^ CYP2D6 Probability: 0–0.5 = non inhibitor; 0.5–1 = inhibitor.

## Supplementary Materials

Supplementary materials can be found at http://www.mdpi.com/1422-0067/17/8/1221/s1.
