# Supplementary material for: Synthesis, Crystal Study, and Anti-Proliferative Activity of Some 2-Benzimidazolylthioacetophenones towards Triple-Negative Breast Cancer MDA-MB-468 Cells as Apoptosis-Inducing Agents
Source: Int J Mol Sci. 2016 Jul 29;17(8):1221. doi: 10.3390/ijms17081221 (PMC5000619; doi:10.3390/ijms17081221)
Supplement: Supplementary file 1 [file ijms-17-01221-s001.pdf]

Supplementary Materials: Synthesis, Crystal Study, and Anti-Proliferative Activity of Some 2-Benzimidazolylthioacetophenones towards Triple-Negative Breast Cancer MDA-MB-468 Cells as Apoptosis-Inducing Agents

Hatem A. Abdel-Aziz, Wagdy M. Eldehna, Hazem Ghabbour, Ghada H. Al-Ansary, Areej M. Assaf and Abdullah Al-Dhfyan

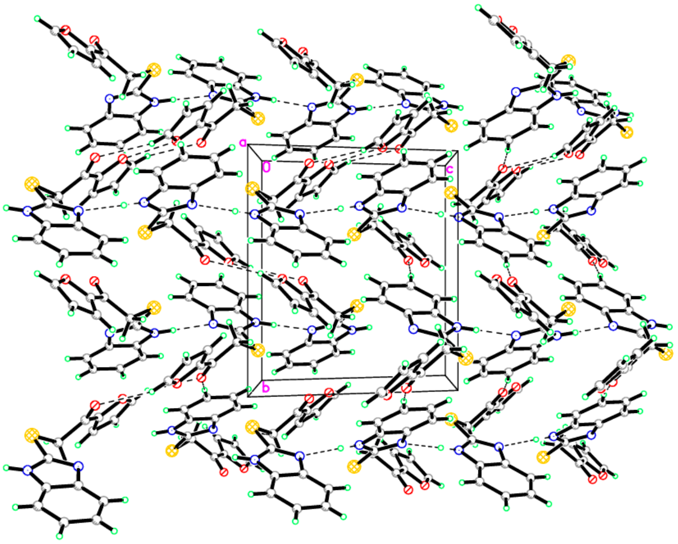

Figure S1. Molecular packing of compound **5v** viewed hydrogen bonds, which are drawn as dashed lines making a network structure.

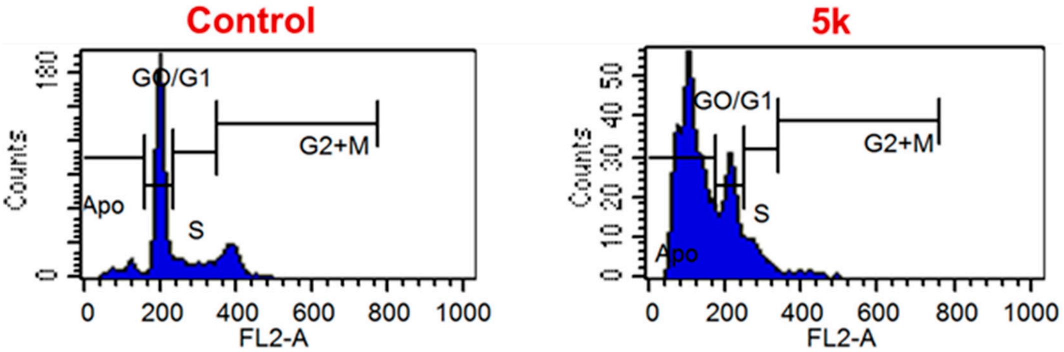

Figure S2. DNA-flow cytometry analysis for MDA-MB-468 cells treated with compound **5k** for 24 h at its IC_{50} concentration. The experiment was done in triplicate.

Table S1. Hydrogen-bond geometry (Å, °) for compound **5v**.

D—H···*A*	*D*—H	H···*A*	*D*···*A*	*D*—H···*A*
N2—H2B···N1 ^{i}	0.8800	2.0000	2.867 (5)	169.00
C3—H3A···O2 ^{ii}	0.9500	2.5700	3.359 (6)	141.00
C11—H11A···O2 ^{iii}	0.9500	2.4700	3.411 (7)	170.00

Symmetry codes: ^{i} x, −y + 1/2, z − 1/2; ^{ii} −x + 1, −y + 1, −z + 2; ^{iii} −x + 2, y − 1/2, −z + 3/2.
